# Reply to familial dysfunction and cognitive decline in aging: the “health, wellbeing, and aging” (SABE) longitudinal population-based study

**DOI:** 10.1590/1980-5764-DN-2024-0146R

**Published:** 2024-12-13

**Authors:** Diego Ferreira da Silva, Juliana Nery Souza-Talarico, Jair Licio Ferreira Santos, Yeda Aparecida de Oliveira Duarte

**Affiliations:** 1 Universidade de São Paulo, Faculdade de Enfermagem, São Paulo SP, Brazil.; 2 University of Iowa, School of Nursing, Iowa City, LA, USA.; 3 Universidade de São Paulo, Faculdade de Medicina de Ribeirão Preto, Ribeirão Preto SP, Brazil.; 4 Universidade de São Paulo, Faculdade de Saúde Pública, São Paulo SP, Brazil.

Dear Editor,

We appreciate the feedback provided by Lopes et al. regarding our recent publication titled “Family dysfunction and cognitive decline in aging: the ‘Health, Wellbeing, and Aging’ (SABE) longitudinal population-based study.” Engaging in constructive dialogue about methodological choices is essential for advancing our understanding in this field, and we thank the authors for their insights.

The authors highlight the cognitive decline measured by three psychometric instruments and the importance of using multivariate analyses, such as Poisson or Cox regression, to assess the effect of family dysfunction on cognitive decline. We understand their concerns regarding using logistic regression (RL) and the potential biases it introduces, including inflated confidence intervals and increased risks of Type II errors. However, our methodology was chosen based on factors that balance both statistical rigor and the practicalities of our data.

While we agree that Poisson or Cox regression can provide valuable insights and allow for estimating relative risks or hazard ratios, it is crucial to address the specific conditions under which these regression models are appropriately applied. Poisson and Cox regression have specific variable requirements, which do not align with the nature of our dataset^1,2^.

Poisson regression is ideal for modeling independent count data within a fixed period. Cox regression models the time until an event occurs, typically understood as survival time^1,2^. The applicability of Cox regression requires clear definitions of event occurrence and time-to-event data^1,2^. In our study, the data available does not robustly support a diagnosis-based analysis as an outcome. SABE data does not include cognitive diagnosis, and the available cognitive and functional available variables cannot support consensus diagnosis. Moreover, while we measure cognitive decline, we have not defined ‘events’ occurring at specific time points for each participant. Cognitive decline is observed continuously rather than as discrete events occurring over time, thus not fulfilling the conditions for appropriate Cox modeling.

We appreciate the authors’ engagement with our study and welcome the discussion surrounding our methodological considerations. A robust analytical approach is pivotal for advancing research in this area, and we remain open to collaborative explorations toward improved methodologies in our future work.

Thank you for your time and consideration.
